# Supplementary material for: Room-Temperature Cholesteric Liquid Crystals of Cellulose Derivatives with Visible Reflection
Source: Polymers (Basel). 2022 Dec 29;15(1):168. doi: 10.3390/polym15010168 (PMC9824885; doi:10.3390/polym15010168)
Supplement: Supplementary file 1 [file polymers-15-00168-s001.zip › polymers-2093326-supplementary.pdf]

Supplementary Materials

Room-Temperature Cholesteric Liquid Crystals of Cellulose Derivatives with Visible Reflection

Yuki Ogiwara, Tatsuya Suzuki, Naoto Iwata and Seiichi Furumi*

Department of Chemistry, Graduate School of Science, Tokyo University of Science, 1-3 Kagurazaka, Shinjuku, Tokyo 162-8601, Japan.
* Correspondence: furumi@rs.tus.ac.jp; Tel.: +81-3-3260-4271

1. Supplementary Figures

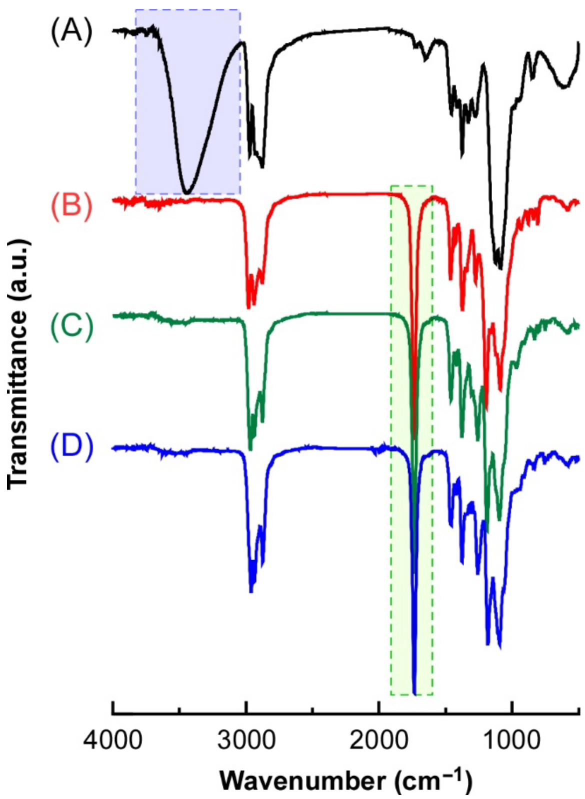

Figure S1. Attenuated total reflection (ATR) FT-IR spectra of pristine HPC (A), HPC-Pr/C7 (B), HPC-Bu/C7 (C), and HPC-Pe/C3 (D). A broad peak of the O-H stretching vibration at 3100–3600 cm^{-1}, highlighted in purple of Figure S1A, disappeared after the esterification of pristine HPC to yield HPC-Pr/C7, HPC-Bu/C7, and HPC-Pe/C3, as shown in Figures S1B, C, and D. As highlighted in green of Figures S1B, S1C, and S1D, sharp peaks of C=O stretching vibration at 1700 cm^{-1} were observed for HPC-Pr/C7, HPC-Bu/C7, and HPC-Pe/C3.

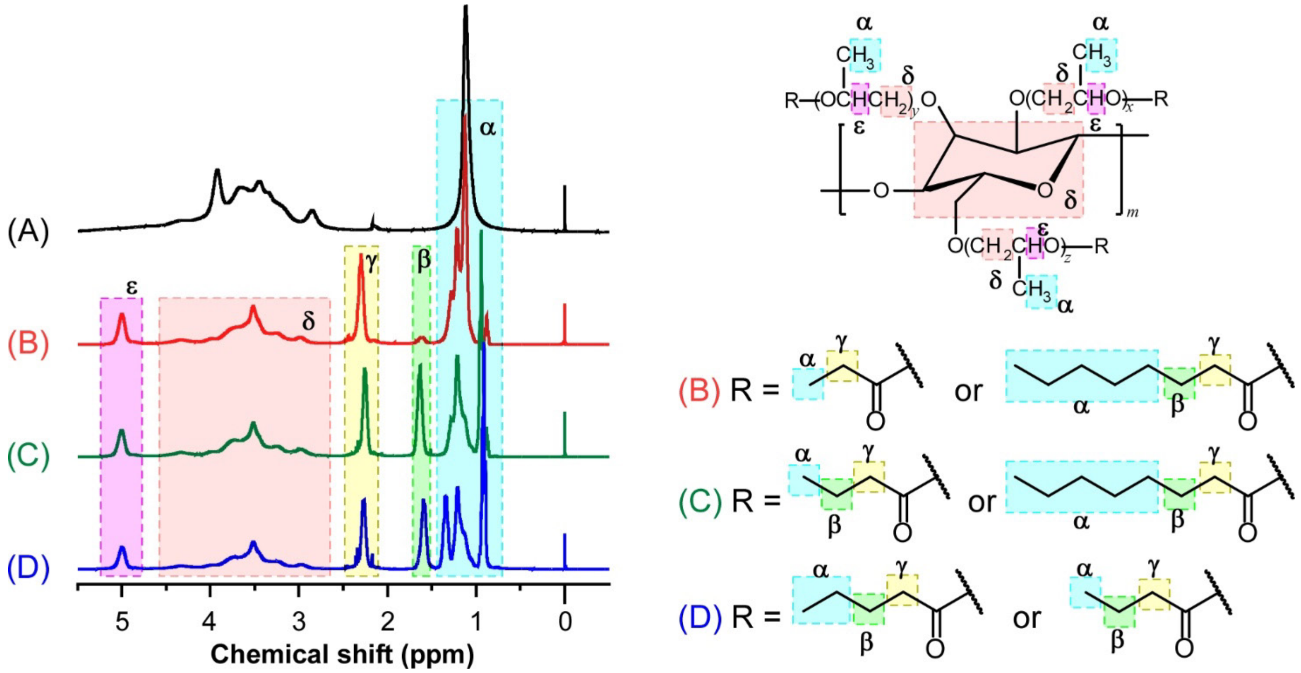

Figure S2. ^{1}H-NMR spectra (left side) and assignments of α−ε peaks (right side) of pristine HPC (A), HPC-Pr/C7 (B), HPC-Bu/C7 (C), and HPC-Pe/C3 (D). As highlighted in blue, the peak α at 0.9−1.4 ppm is assigned to the terminal methyl protons of the alkanoyl groups or hydroxypropyl groups and some methylene protons in the alkanoyl groups. As highlighted in green, the peak β at 1.6−1.7 ppm is assigned to the protons of the methylene groups bonded to the carbon at position two of the alkanoyl groups. As highlighted in yellow, the peak γ at 2.2−2.5 ppm is assigned to the protons adjacent to carbonyl groups. As highlighted in red, the peak δ at 2.6−4.6 ppm is assigned to protons in anhydroglucose monomer unit and methylene groups in hydroxypropyl groups. As highlighted in purple, the peak ε at 4.8−5.2 ppm is assigned to the methine protons in hydroxypropyl groups whose hydroxy groups are substituted with alkanoyl groups.

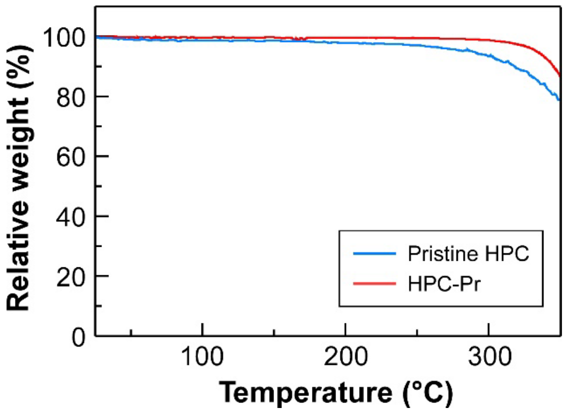

Figure S3. Thermal gravimetry analysis (TGA) curves of pristine HPC (blue line) and HPC-Pr (red line).

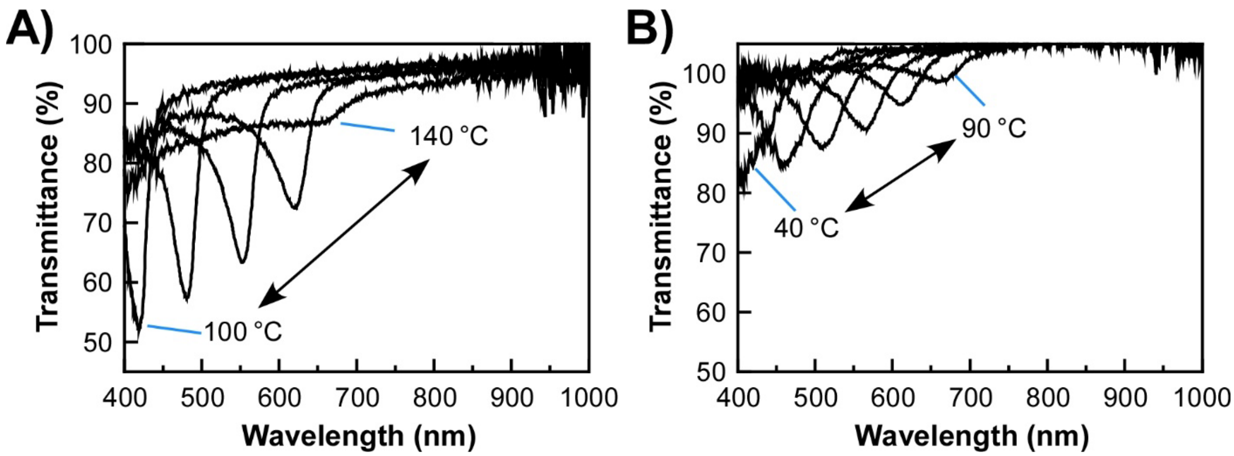

Figure S4. Transmission spectral changes of HPC-Pr (A) and HPC-Bu (B) upon heating process at intervals of 10 °C. The temperature was swept in the range of 100−140 °C for HPC-Pr and of 40−90 °C for HPC-Bu.

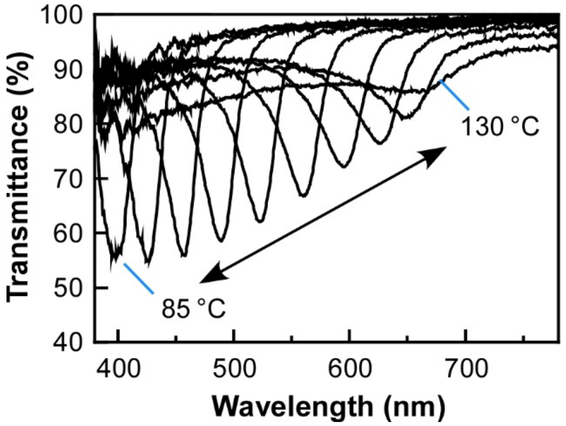

Figure S5. Transmission spectral changes of HPC-Pr/C5 upon heating process from 85 °C to 130 °C at intervals of 5 °C.

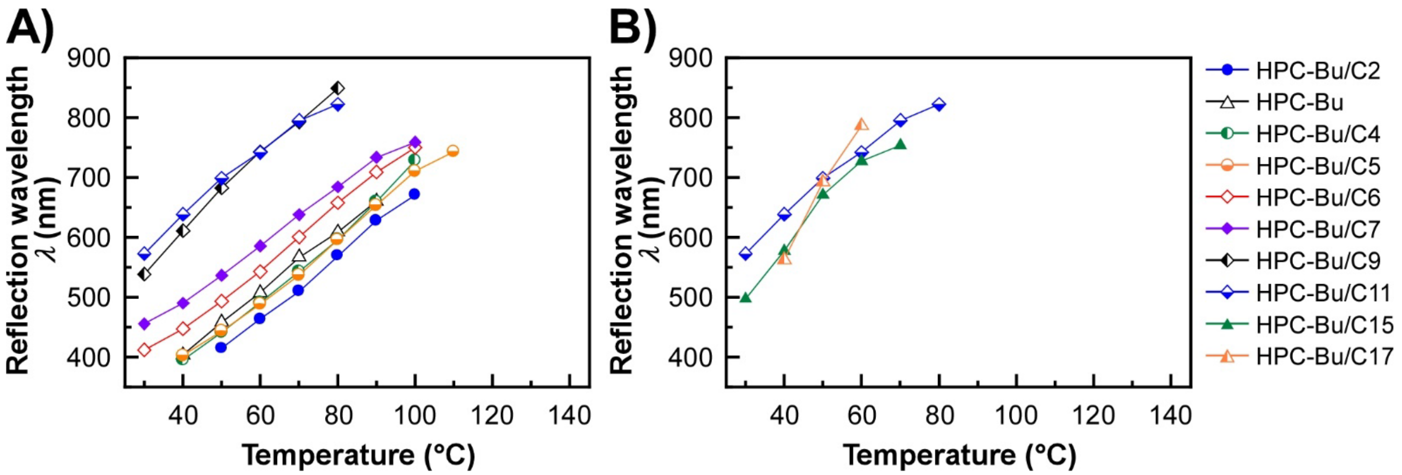

Figure S6. Temperature dependences of reflection peak wavelengths of HPC-Bu/C*n*. (A) HPC-Bu (*n* = 3) and HPC-Bu/C*n* (*n* = 2, 4, 5, 6, 7, 9, and 11). The region of temperature at which HPC-Bu/C*n* reflects visible light shifted toward the lower temperature range as *n* increased. (B) HPC-Pr/C*n* (*n* = 11, 15, and 17). The region of temperature at which HPC-Bu/C*n* reflects visible light shifted toward the higher temperature range as *n* increased. The plots of HPC-Bu/C13 were omitted because HPC-Bu/C13 did not exhibit any reflection peaks, as will be seen in Figure S8.

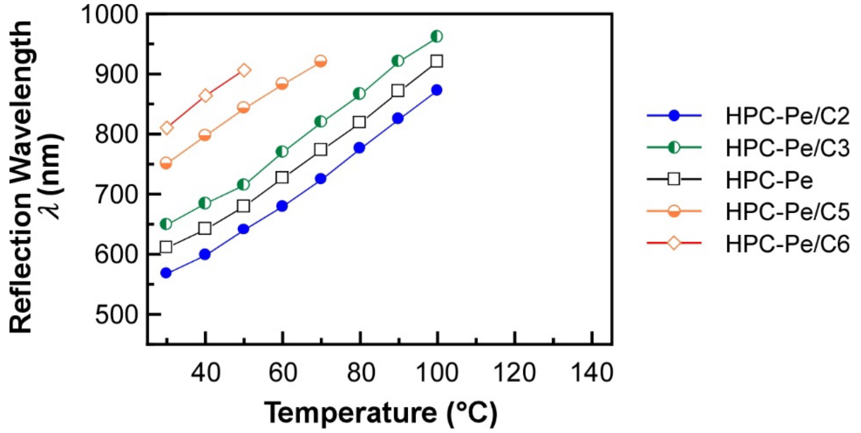

Figure S7. Temperature dependences of reflection peak wavelengths of HPC-Pe (*n* = 4) and HPC-Pe/C*n* (*n* = 2, 3, 5, and 6). The region of temperature at which HPC-Pe/C*n* reflects visible light shifted toward the lower temperature range as *n* increased.

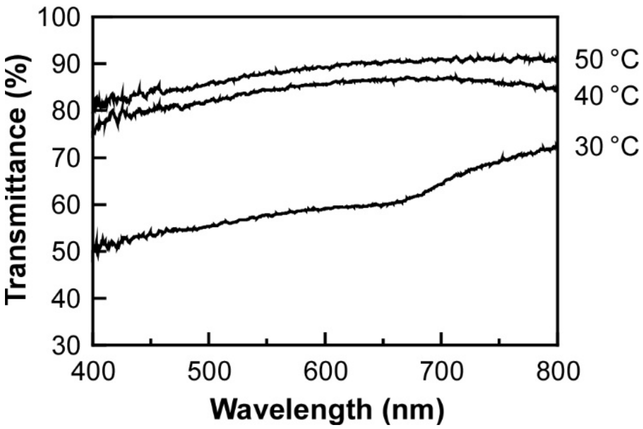

Figure S8. Transmission spectra of HPC-Bu/C13 heated at 30 °C, 40 °C, and 50 °C. HPC-Bu/C13 did not exhibit any reflection peaks.

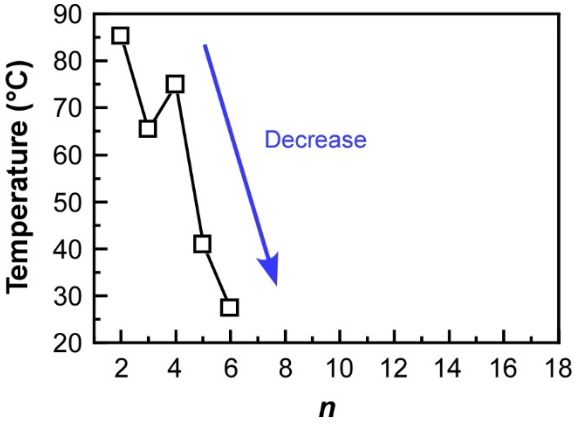

Figure S9. Temperatures at which HPC-Pe (n = 4) and HPC-Pe/Cn showed a reflection peak at 800 nm.

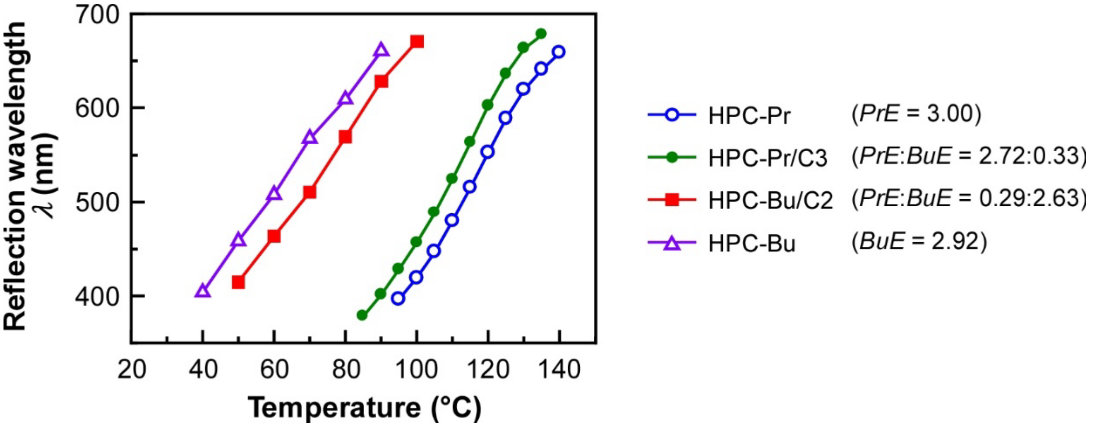

Figure S10. Temperature dependences of the HPC derivatives possessing propionyl groups, butyryl groups, or both with different degrees of substitution.

2. Mathematical Derivation of *XE* and *CnE* of HPC-X/C*n*

The values of XE and CnE of HPC-X/Cn were determined by the integration values of ^{1}H-NMR spectra. Here, X is abbreviated as a major substituted alkanoyl group (Pr, Bu, or Pe), and C*n* stands for the other substituted alkanoyl group (*n* = 2–17). The values of *XE* and *CnE* are the number of the hydroxy groups substituted by alkanoyl chlorides per an anhydroglucose unit. The ^{1}H-NMR spectra of some HPC derivatives (HPC-Pr/C7, HPC-Bu/C7, and HPC-Pe/C3) and the attribution of each peak are available in Figure S2. This procedure is referred to our previous study [1].

2.1. Deviation of *PrE* and *CnE* of HPC-Pr/C*n*

Here, the detailed procedure to determine the values of HPC-Pr/C7 is given. The ^{1}H-NMR spectrum of HPC-Pr/C7 is shown in Figure S2B. The sum of the number of protons per anhydroglucose unit of the HPC-Pr/C7 is expressed by the following equation:

$$7 + 6MS + 5PrE + 15 \times C7E \quad \text{(S1)}$$

where MS is the average number of hydroxypropyl groups per anhydroglucose unit determined to be 3.97 in the Experimental Section, and the coefficients 5 and 15 for *PrE* and *C7E*, respectively, are the number of protons that each alkanoyl group has. Here, we defined the integrated value of the methine proton peak (the peak ε in Figure S2B) as ε and that of the methylene proton peak at 1.6–1.7 ppm, assigned to the protons of the methylene groups bonded to the carbon at position 2 of the alkanoyl groups (the peak β in Figure S2B) as β, and the sum of integrated values of all peaks attributed to HPC derivatives as W. Using the ratio of the number of protons, the following equations are obtained.

$$\frac{\varepsilon}{W} = \frac{PrE + C7E}{7 + 6MS + 5PrE + 15 \times C7E} \quad \text{(S2)}$$

$$\frac{\beta}{W} = \frac{2 \times C7E}{7 + 6MS + 5PrE + 15 \times C7E} \quad \text{(S3)}$$

By solving the Equations (S2) and (S3), the Equations (S4) and (S5) are obtained as follows.

$$PrE = \frac{(7 + 6MS)(\beta - 2\varepsilon)}{6\beta + 10\varepsilon - 2W} \quad \text{(S4)}$$

$$C7E = \frac{\beta(7 + 6MS + 5PrE)}{2W - 15\beta} \quad \text{(S5)}$$

In this way, *PrE* and *C7E* can be determined. Generalizing these calculation procedures for HPC-Pr/C*n*, the Equations (S6) and (S7) are rearranged as follows.

$$PrE = \frac{(7 + 6MS)(\beta - 2\varepsilon)}{(n - 1)\beta + 10\varepsilon - 2W} \quad \text{(S6)}$$

$$CnE = \frac{\beta(7 + 6MS + 5PrE)}{2W - (2n + 1)\beta} \quad \text{(S7)}$$

2.2. Deviations of *BuE* and *CnE* of HPC-Bu/C*n*

Although almost the same procedure as HPC-Pr/Cn can be used to calculate BuE and CnE for HPC-Bu/Cn, we need to consider the overlap in peak β in Figure S2C. As described in Figure S2C, peak β in the ^{1}H-NMR spectrum of HPC-Bu/Cn is attributed to the protons of the methylene groups at the 2-position of the butyryl group and the other minor alkanoyl groups. To separate only the integrated value of the peak due to the butyryl group from that of peak β, we use the product of the alkanoyl chloride preparation molar ratio and β, defined as β'. For example, in the case of HPC-Bu/C7, β' is defined as in the Equation (S8), using the preparation molar ratio shown in Table 2.

$$\beta' = \frac{5.1}{5.1 + 0.3} \times \beta \tag{S8}$$

By introducing β' into equations instead of β, BuE and CnE of HPC-Bu/Cn can be calculated by the same procedure of HPC-Pr/Cn, as shown in the Equations (S9) and (S10).

$$\frac{\varepsilon}{W} = \frac{BuE + CnE}{7 + 6MS + 7BuE + (2n + 1)CnE} \tag{S9}$$

$$\frac{\beta'}{W} = \frac{2CnE}{7 + 6MS + 7BuE + (2n + 1)CnE} \tag{S10}$$

By the Equations (S11) and (S12), which are obtained by the Equations (S9) and (S10), BuE and CnE of HPC-Bu/Cn can be determined.

$$BuE = \frac{(7 + 6MS)(\beta' - 2\varepsilon)}{(2n - 6)\beta' + 14\varepsilon - 2W} \tag{S11}$$

$$CnE = \frac{\beta'(7 + 6MS + 7BuE)}{2W - (2n + 1)\beta'} \tag{S12}$$

2.3. Deviations of *PeE* and *CnE* of HPC-Pe/C*n*

The value of *PeE* and *CnE* of HPC-Pe/C*n* can be determined by the almost same procedure as HPC-Bu/C*n*. In the case of HPC-Pe/C*n*, the Equations (S13) and (S14) can be obtained by introducing γ', defined as the product of the preparation molar ratio of alkanoyl chlorides and γ, where γ is the integrated value of the peak at 2.2–2.5 ppm (peak γ in Figure S2D).

$$\frac{\varepsilon}{W} = \frac{PeE + CnE}{7 + 6MS + 9PeE + (2n+1)CnE} \tag{S13}$$

$$\frac{\gamma'}{W} = \frac{2CnE}{7 + 6MS + 9PeE + (2n+1)CnE} \tag{S14}$$

By solving the Equations (S13) and (S14), *PeE* and *CnE* of HPC-Pe/C*n* can be obtained, as shown in the Equations (S15) and (S16).

$$PeE = \frac{(7 + 6MS)(\gamma' - 2\varepsilon)}{(2n-8)\gamma' + 18\varepsilon - 2W} \tag{S15}$$

$$CnE = \frac{\gamma'(7 + 6MS + 9PeE)}{2W - (2n+1)\gamma'} \tag{S16}$$

3. Supplementary Reference

1. Ishizaki, T.; Uenuma, S.; Furumi, S. Thermotropic Properties of Cholesteric Liquid Crystal from Hydroxypropyl Cellulose Mixed Esters. *Kobunshi Ronbunshu* **2015**, *72*, 737–745.
